# Reviewed article: Feitosa YS, Cabral BVB, Lima GS, Gomes EB, Sousa GJB, Moreira TMM, Pereira MLD. Surgical treatment of diabetic foot: a spatial analysis, Ceará, Brazil, 2016-2021. Epidemiol Serv Saude. 2026:35;e20240755

**DOI:** 10.1590/S2237-96222026v35e20240755.b

**Published:** 2025-12-19

**Authors:** Betine Pinto Moehlecke Iser

**Affiliations:** 1 Universidade do Sul de Santa Catarina, Tubarão, SC, Brazil Universidade do Sul de Santa Catarina Tubarão SC Brazil

## First round comments

Caros autores

Nosso corpo editorial considerou seu estudo de boa qualidade e passível de publicação, mediante alterações.

No entanto, cabem alguns ajustes, em especial referente às normas da RESS, além do aprimoramento do texto.

Sumarizo a seguir itens importantes a serem revistos:

1. Na introdução, sugere-se finalizar com a frase de objetivo do estudo, retirando a sentença das linhas 57-60 da página 2 e linhas 3 e 5 da página 3.

2. A descrição dos métodos está confusa, e pode ser aprimorada seguindo os itens indicados no STROBE

/ RECORD e normas da revista.

2.1 Como foram tratados os dados de dois sistemas distintos - SIH e SISAB? Entende-se que o SISAB foi utilizado como referência para captar o número de pessoas com DM no Estado. Sabe-se que a informação sobre a população com DM, ainda mais unicamente nos dados da atenção básica, é pouco precisa, o que pode ser considerada uma limitação importante do estudo, a qual precisa ser indicada e contextualizada.

2.2 Quanto à dispensa de apreciação ética em virtude do uso de dados secundários de domínio público, orienta-se a citação da mais recente Resolução que aborda o tema: Resolução CNS 674 de 2022 (Artigo 26 Incisos II, III e V). Tal informação deve constar apenas na folha de rosto.

2.3 Indicar o que significa a “análise puramente espacial”.

3. Resultados

3.1 Na tabela 1, que apresenta a caracterização das internações por diabetes mellitus, cabe ressaltar que o uso da proporção é influenciado pelo tamanho populacional de cada categoria. Assim, as maiores proporções na capital Fortaleza são esperadas, pelo maior porte populacional, e talvez por ser referência para casos mais complicados. Essa influência deve ser incluída na discussão.

3.2 Sugere-se colocar o valor-p real originado das análises, e não apenas se p-valor < ou > 0,05.

3.3 Atente-se para a necessidade de ajustes nas figuras para melhor visualização dos dados.

4. Discussão: de modo geral precisa ser aprimorada, evitando a citação de dados da literatura sem a contextualização com os achados do estudo. As limitações precisam ser melhor descritas, indicando a sua possível influência nos resultados. Os dados do numero de casos de diabetes em cada município, denominador da taxa apresentada, são influenciados por diversos fatores que são diferentes em cada local e precisam ser apontados.

4.1 Primeiro parágrafo da discussão deve sumarizar os principais resultados do estudo, e não indicar dados

da literatura.

4.2 Evitar siglas desnecessárias, como PND.

4.3 Página 7/16, linhas 37 a 42 são trechos que apresentam ideias repetidas e fora de contexto.

Por favor, avalie cuidadosamente as revisões e envie uma versão reformulada no manuscrito para nova avaliação.

Agradecemos seu interesse pelo RESS.

Recommendation: Major revision

## Second round comments

Caros autores

Agradecemos o envio da sua versão revisada. Não encontramos, porém, a resposta item a item das questões levantadas pelo corpo editorial. Em análise da nova versão, a partir dos destaques no texto, encontramos pontos que precisaram ser esclarecidos e/ou aprimorados, detalhados a seguir:

1.Verificar na introdução, 2º parágrafo, a frase das linhas 6-12, pois não está indicado o verbo da ação 

2. Métodos - indica-se como um estudo de séries temporais, e como unidade de análise os municípios do Ceará. Faz-se necessário indicar o estudo, de acordo com o título, como também análise espacial, e citar a unidade de análise da tendência temporal (anos). Quanto a isto, sugere-se avaliar as indicações da revisão estatística, em anexo.

3. Nos resultados, aparece no título a denominação “Tratamento do pé diabético complicado” - o que seria esse complicado? No texto dos métodos, indica-se como “tratamento cirúrgico”. Recomenda-se conceituar e/ou padronizar os termos utilizados.

3.1. Na figura 1, sugere-se indicar o mapa do Ceara a partir da contextualização das Américas- Brasil para melhor entendimento de leitores de outros locais

3.2. Figura 2, é preciso indicar as unidades em que a variável é plotada. Ainda não está conforme as recomendações.

3.3 Figura 4A, na legenda, os valores referem-se ao RR?

4. Discussão: Rever a pontuação e uso de virgulas, na página 7/20, linhas 57-60

5. Quanto ao item de Disponibilidade de dados, deve-se indicar o acesso aos dados específicos do artigo - já trabalhados, indicadores calculados, indicando inclusive uma possível relação entre SIH e SISAB - e não os dados brutos acessados via DATASUS, cujo acesso é público.

Além disso, nossa revisão estatística especializada apontou questões importantes a serem sanadas na seleção dos métodos estatísticos utilizados, os quais devem ser cuidadosamente avaliados (em anexo).

Por favor, avaliem atentamente e nos enviem uma resposta item a item das sugestões, para facilitar o

processo de revisão.

Recommendation: Major revision

## Third round comments

Caros autores

Agradecemos seu esforço na revisão cuidadosa às recomendações do corpo editorial. Apontamos ainda alguns ajustes a serem feitos, alguns não alterados adequadamente após a revisão anterior:

1. Introdução: 2º parágrafo (página 2, linhas 13-17): Sugere-se evitar o uso da escrita indireta na frase “Esse aumento das complicações graves associadas ao diabetes mellitus, como as úlceras podálicas, (verbo que relacione ao aumento? ) que, quando não diagnosticadas e tratadas precocemente, podem evoluir para infecções e amputações de membros inferiores”.

2. Métodos: ajustes realizados.

3. Resultados: Página 6, linhas 48-49, encontra-se que “Os resultados deste estudo evidenciam que os casos de tratamento cirúrgico do pé diabético no Ceará mantiveram-se estáveis ao longo do período analisado.”

No entanto, esse resultado estaria vinculado a análise de tendência temporal, a qual foi suprimida do manuscrito, portanto deve ser suprimido ou alterado.

4. A partir desse ponto (página 6, linha 51), encontram-se interpretações dos resultados e a contextualização com a literatura, parágrafos que devem ser realocados para a seção de discussão do manuscrito (páginas 7-9), seção esta que não está indicada no manuscrito. Ainda, deve-se cuidar com o limite de caracteres do manuscrito, e evitar repetição de idéias, tais como as citações, nas páginas 8 e 9, sobre as disparidades econômicas (Indice de Desenvolvimento Humano) e de acesso aos serviços de saúde, que poderiam ser sumarizadas.

5. Na página 7, frase da linhas 14-15 “associado a fatores culturais relacionados ao sexo masculino que, historicamente, atribuem menos importância ao autocuidado na vida adulta” idealmente deve ser referenciada.

6. Nas figuras 2 e 3, ainda encontra-se no título o termo “tratamento de pé diabético complicado”, sem definição deste. Sugere-se conceituar o termo previamente, na seção métodos, e/ou destacar nos resultados, quando da citação dos dados das figuras. Ainda não foram indicadas as unidades das medidas (taxas) indicadas nas figuras 2 A e B.

Recommendation: Minor revision

## Fourth round comments

Caros autores

Os ajustes necessários foram realizados, no entanto, em atendimento às normas da RESS, o parágrafo das limitações deve ser deslocado como 2º parágrafo, logo após a sumarização dos resultados principais (1º parágrafo da discussão). Aproveitamos para pontuar outros pequenos ajustes no texto, como a indicação da Resolução específica que trata da dispensa de avaliação ética para estudos com dados de domínio público (Resolução 674/2022), além da revisão ortográfica e de pontuação. Por favor, vejam o documento com comentários diretamente no texto, em anexo, para facilitar a identificação dos ajustes necessários.

Recommendation: Minor revision

